# Epidemiological and Clinical Characteristics of Non-Typhoidal *Salmonella* Bloodstream Infections in Central Israel: A Case-Control Study

**DOI:** 10.3390/microorganisms10101942

**Published:** 2022-09-30

**Authors:** Yael Israel, Khitam Muhsen, Assaf Rokney, Amos Adler

**Affiliations:** 1 Department of Epidemiology and Preventive Medicine, School of Public Health, Sackler Faculty of Medicine, Tel Aviv University, Tel Aviv 6139001, Israel; 2 Public Health Laboratories-Jerusalem (PHL-J), Public Health Services (PHS), Ministry of Health (MOH), Jerusalem 9134302, Israel; 3 Clinical Microbiology, Tel Aviv Sourasky Medical Center, Tel Aviv 6423906, Israel

**Keywords:** non-typhoidal *Salmonella*, bloodstream infections, immunocompromised

## Abstract

Non-typhoidal *Salmonella* (NTS) infection continues to be a significant cause of morbidity. In addition to gastroenteritis (GE), NTS may cause bloodstream infections (BSI). Our goals were to characterize the demographics, clinical characteristics and outcome of NTS-BSI in central Israel. The study was a retrospective, case-control study conducted at the Tel Aviv Sourasky Medical Center between 2001–2018. Cases with NTS-BSI were matched by age and compared with two control groups, hospitalized patients with NTS-GE and patients with *E. coli* BSI. The NTS-BSI group included 34 patients who were compared with 69 and 68 patients in the NTS-GE and *E. coli* BSI groups, respectively. In the NTS-BSI group, the median age was 59 years, with 20% of patients below 20 years of age. Diarrhea was less common in NTS-BSI patients compared with NTS-GE: 53% vs. 80% (*p* < 0.01). Compared with NTS-GE patients, NTS-BSI patients had a higher rate of recent antimicrobial use: 21% vs. 5.9%, *p* = 0.03, respectively. They also had a slightly higher Charlson Comorbidity Index score, and history of past malignancy and steroid use, but these differences were not statistically significant. Antimicrobial treatment was documented in 30/34 of the NTS-BSI patients vs. 55/69 of the NTS-GE patients (*p* < 0.001). NTS-BSI patients had higher rates of in-hospital death (23% vs. 4%, *p* < 0.01) and a longer length of stay (8 vs. 4 days, *p* < 0.001) compared with NTS-GE. There was no significant difference in the outcome compared with the *E. coli* BSI group. In conclusion, our study found relatively low rates of pediatric cases compared with previous studies in Israel. NTS-BSI patients had slightly higher rates of comorbidities compared with NTS-GE patients, and a similar prognosis to *E. coli* BSI patients.

## 1. Introduction

Non-typhoidal *Salmonella* (NTS) infection is a major cause for morbidity and mortality, especially in sub-Saharan Africa and other parts of the developing world [1]. In addition to gastroenteritis (GE), which is by far the most common infection, NTS may cause a variety of invasive infections, such as bloodstream infections (BSI) or osteomyelitis, especially in patients with HIV and other types of immunodeficiency [2].

Although invasive NTS infection possesses the greatest burden in Africa, there is still substantial risk for invasive NTS infection across the world [1], including cases of drug-resistant infections [3]. Despite a steady decline in the incidence of Salmonellosis in Israel [4], the incidence of invasive NTS infections is relatively high compared with other high-income countries [1]. This difference might be due to a variety of causes, including a relatively high number of children, climate, and food hygiene practices. 

The clinical characteristics of NTS-BSI in Israel have been described in a single-center study in Jerusalem [5]. In that study, the majority of patients were children (54%), and NTS-BSI was associated with a higher rate of comorbidities compared with patients with NTS-GE. 

The Tel Aviv district is located in the center of Israel; it is characterized by a western urban lifestyle and a unique population structure. The population of the Tel Aviv district is demographically different to the Jerusalem population, with a larger elderly population, higher socioeconomic ranking, and a lower rate of Arab residents and ultraorthodox Jewish residents, two groups that are characterized by high-fertility and living in crowded households. Similar socio-demographic factors were shown to be related to Salmonella infections in high-income countries such as Canada, Europe and the United States [6,7,8]. The incidence of salmonellosis in Israel was shown to vary geographically and according to rural-urban residential areas and population groups [9]. Hence, the characteristics of NTS-BSI were identified in the Tel Aviv population as a model of urban settings, and the potential correlates, clinical characteristics and outcomes, such as length of hospital stay, transfer to intensive care unit (ICU) and in-hospital mortality were explored. 

## 2. Methods

### 2.1. Setup and Study Design

The Tel-Aviv Sourasky Medical Center (TASMC) is a 1500-bed, tertiary care center and is the only general hospital serving the population of Tel-Aviv, Israel. This was a retrospective case-control study. Inclusion criteria included all patients admitted to TASMC and who were diagnosed with NTS-BSI (excluding *S. typhi* but also *S. paratyphi*), between 2001 and 2018. Cases were matched by age (+/− 5 years) with a 1:2 ratio to two control groups: (1) hospitalized patients with NTS-GE, to discern specific risk factors for NTS-BSI; (2) hospitalized patients with *E. coli* BSI, to examine differences in outcome between the two BSIs. The former comparison is expected to enhance the understanding regarding the risk of NTS-BSI in patients who get *Salmonella* infection, while the latter comparison is expected to shed light on the difference between the two BSI beyond the propensity of bacteremia. Non-hospitalized patients were excluded.

### 2.2. Microbiological Methods and Definitions

Stool and blood cultures were processed according to American Society of Microbiology protocols [10]. Blood cultures were analyzed using the BACT/ALERT^®^ 3D^®^ system (2001–July 2017) or the VIRTUO^®^ system (August 2017–2018) (both by bioMerieux, Marcy l’Etoile, France). Bacterial identification of NTS was carried out using biochemical tests, Salmonella polyvalent O antigen testing and either the VITEK-2 (before 2016) or the VITEK-MS^®^ MALDI- (2016–2018) ToF identification systems (bioMerieux, Marcy l’Etoile, France). Antimicrobial susceptibility testing (AST) was carried out using the VITEK2^®^ system (bioMerieux, Marcy l’Etoile, France). AST breakpoints were interpreted and changed according to CLSI criteria [11]. Susceptibility to quinolones was screened by disk diffusion to Nalidixic acid until 2016, followed by adjustment of the VITEK2^®^ system AST card to the lower MIC breakpoints for ciprofloxacin. NTS isolates were sent to the National *Salmonella* Reference Laboratory at the Ministry of Health for serovar identification.

### 2.3. Data Collection and Analysis

Patients in each group were identified using the Microbiology Laboratory records. Patient data were extracted from electronic medical files. Background and current variables included demographic data, the presence of gastrointestinal symptoms, initial laboratory results, background medical conditions including the Charlson Comorbidity Index (CCI) [12], recent (2 weeks) hospitalization and antimicrobial use, microbiological data including the number and type of positive cultures, and data regarding the use of antimicrobial treatment. 

Outcome variables included: (a) total length of stay (LOS); (b) rate of transfer to ICU; (c) in-hospital mortality and (d) recurrent admissions. 

Statistical analysis was performed using SPSS software version 27 (IBM, Armonk, New York, NY, USA). The NTS-BSI group was compared to each of the other two groups separately. Univariate tests included Student’s *t*-test for continuous variables, and the chi-square test for dichotomous and categorical variables.

### 2.4. Ethical Statement 

The study was approved by the Ethics Committee of the TASMC (Number 0071-19-TLV).

### 2.5. Power Calculations

A sample of 34 cases and 68 controls provided a statistical power of 79%, assuming a 50% prevalence of a certain risk factor among the control group and an odds ratio of 3.5. The power calculation was executed using Winpepi software [13].

## 3. Results

### 3.1. Demographic and Clinical Features of Patients with NTS-BSI vs. Control Groups

In the 18 years of the study (2001—2018), we identified 34 cases of NTS-BSI, which were compared to two control groups: NTS-GE (*n* = 69) and *E. coli* BSI (*n* = 68). 

The median age was 59 years, with 20% of patients below 20 years of age (Table 1). Accordingly, the majority of patients were admitted to Internal Medicine wards (71%, 78% and 67% in NTS-BSI, NTS-GE and *E. coli* BSI, respectively), followed by Pediatric wards (23%, 20%, 17% in NTS-BSI, NTS-GE and *E. coli* BSI, respectively). Male patients comprised 64% of the patients and the majority were of Jewish descent; none of the demographic features differed significantly from the other groups.

Gastrointestinal symptoms were less common in NTS-BSI patients compared with the patients with NTS-GE: diarrhea 53% vs. 80% (*p* < 0.01), emesis 19% vs. 39% (*p* < 0.05), as well as fever 45% vs. 63% (*p* = 0.34) in NTS-BSI vs. NTS-GE, respectively (Table 2). Diarrhea and emesis were uncommon in patients with *E. coli* BSI. Baseline mean hemoglobin levels were lower and C-reactive protein levels were higher in NTS-BSI patients compared with NTS-GE patients: 10.8 g/dL vs. 12.7 g/dL, *p* < 0.001, and 134 mg/L vs. 88 mg/L, *p* < 0.05, respectively. There were no differences between the groups in the neutrophil or platelets levels, or in comparison with the *E. coli* BSI group.

Compared with NTS-GE patients, NTS-BSI patients had a slightly higher CCI and history of past malignancy and steroid use, but the differences were not statistically significant. Likewise, these risk factors did not differ in comparison with *E. coli* BSI patients, although they did have a higher rate of recent hospital admissions (12% vs. 1.5%, respectively, *p* = 0.03). NTS-BSI patients had a higher rate of recent antimicrobial use compared with NTS-GE patients: 21% vs. 5.9%, *p* < 0.05, respectively. There were no statistically significant differences in the rates of ischemic heart disease, congestive heart failure, arrhythmia, diabetes, chronic lung disease, chronic renal insufficiency, HIV infection or solid organ transplantation between the three groups (data not shown). 

### 3.2. Microbiological Features of NTS-BSI Isolates

The duration of NTS-BSI was relatively short; in most cases (28/34, 82%) had one positive blood culture, five had two, and only one case had three positive cultures. Patients with NTS-BSI had concurrent positive stool or urine cultures in eight and four cases, respectively.

The serovar was identified and documented in 18 cases and included *Enteritidis* (*n* = 13), *Infantis* (*n* = 2), *Bredeney* (*n* = 2) and *Havana* (*n* = 1). Susceptibility rates to ampicillin, ceftriaxone, ciprofloxacin and trimethoprim-sulphamethoxazole were 91%, 100%, 88% and 97%, respectively.

### 3.3. Treatment and Outcome of NTS-BSI Patients

Antimicrobial treatment was documented in 30/34 of the NTS-BSI patients. Ceftriaxone was the most common antimicrobial agent, and was used either as the first (*n* = 16) or second (*n* = 6) antimicrobial choice in 22/30 patients (73%). Other agents included piperacillin-tazobactam (*n* = 4), ciprofloxacin or levofloxacin (*n* = 2), cefuroxime or cefepime (*n* = 1 each). Antimicrobial treatment was documented in 55/69 and 63/68 of patients with NTS-GE and *E. coli* BSI, respectively (*p* < 0.001). 

NTS-BSI patients had a higher rate of in-hospital death (23% vs. 4%, *p* = 0.008, respectively), and a longer length of stay (8 vs. 4 days, *p* < 0.001, respectively), compared with NTS-GE patients (Table 3). There was no significant difference in outcome measures compared with the *E. coli* BSI group. Rates of ICU transfer and recurrent admissions were low in all groups.

## 4. Discussion

The incidence of *Salmonella* infection in Israel is relatively high compared with other developed countries [4]. Although the incidence of both intestinal and extra intestinal salmonellosis had declined in the first decade of the millennia [4,14], recent reports have shown a reversal of this trend, with an increasing number of cases due to *S.* Enteritidis [15]. Hence, as salmonellosis continues to pose a significant burden on public health in Israel, it is important to understand the risk factors for invasive infection in different populations. 

In the current study in Tel Aviv, the frequency of gastrointestinal symptoms, including diarrhea, was lower in NTS-BSI compared with NTS-GE, 53% vs. 80%, respectively. This rate was similar to a the rate previously reported from Israel (57%) [16], was a higher rate than in a previous study from Britain (18%) [17], and is likely reflecting the gastrointestinal source of most BSI cases in our study. 

As for other baseline characteristics, we found only a few differences in underlying morbidities in patients hospitalized with NTS-GE compared with NTS-BSI. Patients with BSI had higher rates of recent emergency department admission and antimicrobial use. Unlike our previous study which was performed in Jerusalem [5], the CCI score and the rate of steroid use were not significantly higher compared with the other two groups. A possible explanation might be related to the relatively low rate of pediatric patients in our study (20% vs. 54%) [5]. In the pediatric population, patients might still be hospitalized with NTS-GE for supportive care (e.g., parenteral fluids), even without any underlying conditions. Thus, since age matching was done in both, the higher rate of pediatric patients in the Jerusalem study might have been the cause for these differences. 

Another important aspect to consider is that among all patients with NTS-GE, only a small minority of patients are hospitalized. This subset of patients was likely to be sicker, and thus, our study does not reflect the difference in risk factors from the total population of NTS-GE patients.

As expected, the outcome of patients with NTS-BSI was poorer in terms of both in-hospital mortality and length of stay compared with NTS-GE, despite a lower rate of antimicrobial treatment in the latter. The mortality was significant (23%), despite the fact that most cases were treated adequately (ceftriaxone), and was similar to the mortality of *E. coli* BSI (19%). The mortality was comparable to other reports [16,17], although the comparison is limited due to the differences in age range. 

The majority of BSI isolates that were characterized to the serovar level were *S.* Enteritidis (13/18, 72%), although the small number and the long duration of the study hinders meaningful analysis of temporal trends. *S.* Enteritidis was the most common serovar isolated from blood in Israel between 1996–2006 [14], but to a much smaller extent (364/1049, 35%) in comparison with our study. *S.* Enteritidis remained a dominant serovar (first or second) from human sources in Israel throughout the following years, up to 2020 [15]. Most isolates were susceptible to all antimicrobial agents tested. Albeit the susceptibility to ciprofloxacin was high (88%), it does not take into account the changes made in the breakpoint definitions. It is likely that with the current, more stringent definitions [18], the susceptibility rate would be lower. Since treatment with quinolone was attempted in only two cases, it is not likely that this change had significant clinical implications. 

The main limitations in our study are mostly related to the relatively small number of NTS-BSI cases that occurred in our institution, which might have led to difficulties in discerning significant differences between the groups. Also, the comparison to hospitalized NTS-GE patients might have led to the overrepresentation of patients with comorbidities in that group, compared with their share in the community.

## 5. Conclusions

Our study demonstrates the nuanced differences in the epidemiology of NTS-BSI, even within the same country, depending mostly on the age distribution and the frequencies of comorbidities. The recent increase in the incidence of salmonellosis in Israel [15] highlights the need for constant vigilance, since the morbidity of NTS-BSI is substantial, even in the developed world. 

## Figures and Tables

**Table 1:** Demographic and medical characteristics of patients in all three groups.

Variable	NTS ^1^-BSI ^2^ (*n* = 34)	NTS-GE ^3^ (*n* = 69)	*E. coli* BSI (*n* = 68)	*p*-Value
Gender, male, *n* (%)	22 (64)	36 (52)	32 (47)	NS ^4^
Age, median (95% C.I.)	59 (24, 75)	59 (21, 75)	62 (25, 73)	NS ^4^
0–19 years, *n* (%)	7 (20)	17 (25)	14 (21)	
20–59 years, *n* (%)	12 (35)	18 (26)	16 (23)	
≥60 years, *n* (%)	15 (45)	34 (49)	38 (56)	
Ethnicity, Jewish, *n* (%)	30 (88)	65 (100)	56 (84)	<0.05 ^5^
Recent (2 weeks) emergency room visit, *n* (%)	1 (3)	3 (4)	2 (3)	NS ^4^
Recent (2 weeks) hospital admission, *n* (%)	4 (12)	5 (7)	1 (1.5)	<0.05 ^6^
Recent (2 weeks) antimicrobial use, *n*, (%)	7 (21)	4 (5.9)	9 (13.6)	<0.05 ^5^
Charlson Comorbidity Index, mean (SD)	2.35 (2.84)	1.78 (2.36)	2.43 (2.92)	NS
Past malignancy, *n* (%)	8 (24)	9 (13)	18 (27)	NS
Steroid use, *n* (%)	6 (18)	6 (8)	5 (7)	NS

^1^-NTS-non-typhoidal *Salmonella*; ^2^-BSI-bloodstream infection; ^3^-GE-gastroenteritis; ^4^-NS-not significant; ^5^-NTS-BSI vs. NTS-GE; ^6^-NTS-BSI vs. *E. coli* BSI.

**Table 2:** Gastrointestinal symptoms and laboratory findings at presentation.

Variable	NTS ^1^-BSI ^2^ (*n* = 34)	NTS-GE ^3^ (*n* = 69)	*E. coli* BSI (*n* = 68)	*p*-Value
Fever	9 (45)	27 (63)	31 (84)	NS ^4^
Diarrhea	17 (53)	55 (80)	8 (12)	<0.001 ^6^
Emesis	6 (19)	27 (39)	16 (24)	<0.05 ^5^
Mean hemoglobin levels (g/dL)	10.4	12.7	10.75	<0.001 ^5^
C-reactive protein concentration (mg/L)	134	88	147	<0.05 ^5^
Neutrophil ratio (%)	78	78	77	NS ^4^
Platelets count (cpm)	230,000	240,000	228,000	NS ^4^

^1^-NTS-non-typhoidal *Salmonella*; ^2^-BSI-bloodstream infection; ^3^-GE-gastroenteritis; ^4^-NS-not significant; ^5^-NTS-BSI vs. NTS-GE; ^6^-NTS-BSI vs. *E. coli* BSI.

**Table 3:** Outcome measures of patients in all three groups.

Variable	NTS ^1^-BSI ^2^ (*n* = 34)	NTS-GE ^3^ (*n* = 69)	*E. coli* BSI (*n* = 68)	*p*-Value
In-hospital death, *n* (%)	8 (23)	3 (4)	13 (19)	<0.01 ^4^
ICU transfer, *n* (%)	0	0	1 (1.5)	NS
Length of stay, median (95% C.I.)	8 (1, 371)	4 (0, 33)	7 (1, 75)	<0.001 ^4^
Recurrent admission within a year, *n* (%)	1 (3)	2 (3)	1 (2)	NS ^5^

^1^-NTS-non-typhoidal *Salmonella*; ^2^-BSI-bloodstream infection; ^3^-GE-gastroenteritis; ^4^-NTS-BSI vs. NTS-GE; ^5^-NS-not significant.

## Data Availability

The data presented in this study are available on request from the corresponding author.
